# Exploring the Emotional Breastfeeding Experience of First-Time Mothers: Implications for Healthcare Support

**DOI:** 10.3389/fped.2020.00199

**Published:** 2020-05-07

**Authors:** Maria Lorella Giannì, Marta Lanzani, Alessandra Consales, Giovanna Bestetti, Lorenzo Colombo, Maria Enrica Bettinelli, Laura Plevani, Daniela Morniroli, Gabriele Sorrentino, Elena Bezze, Lidia Zanotta, Patrizio Sannino, Giacomo Cavallaro, Eduardo Villamor, Paola Marchisio, Fabio Mosca

**Affiliations:** ^1^Fondazione IRCCS Cà Granda Ospedale Maggiore Policlinico, NICU, Milan, Italy; ^2^Department of Clinical Sciences and Community Health, University of Milan, Milan, Italy; ^3^Dipartimento di Scienze Umane per la Formazione “Riccardo Massa,” Università di Milano-Bicocca, Milan, Italy; ^4^Fondazione IRCCS Cà Granda Ospedale Maggiore Policlinico, S.I.T.R.A. Basic Education Sector, Milan, Italy; ^5^Department of Pediatrics, Maastricht University Medical Center (MUMC+), School for Oncology and Developmental Biology (GROW), Maastricht, Netherlands; ^6^Fondazione IRCCS Ca' Granda Ospedale Maggiore Policlinico, Milan, Italy; ^7^Department of Pathophysiology and Transplantation, University of Milan, Milan, Italy

**Keywords:** breastfeeding experience, breastfeeding care, emotional experience, first-time mothers, healthcare support

## Abstract

**Background:** Among breastfeeding determinants, the unique emotional breastfeeding experience has been poorly explored. The present study aimed to investigate the emotional breastfeeding experience in a cohort of first-time mothers.

**Materials and methods:** We conducted a prospective observational study that enrolled primiparas having delivered singleton healthy term newborns, and exclusively breastfeeding at hospital discharge. At 3 months post-delivery mothers accessed an online questionnaire investigating their emotional breastfeeding experience. The chi-squared test was used to assess the association between the feelings experienced during breastfeeding and feeding outcomes at 3 months.

**Results:** Out of the 421 enrolled mothers, 273 (65%) completed the questionnaire. At 3 months post-delivery exclusive breastfeeding was reported by a 66% of mothers, a 19% reported complementary feeding, and a 15% of mothers reported exclusive formula feeding. Breastfeeding experience was described as positive by 62% of mothers although breastfeeding difficulties were reported by 80% of the mothers. The mothers that had experienced fear, sadness, anger or concern during breastfeeding showed a significant higher exclusive formula feeding rate at 3 months post-delivery than those who did not (25.5 vs. 12.8%, *p* = 0.021; 28.6 vs. 13.4%, *p* = 0.02; 40 vs. 13.4%, *p* = 0.005; 20.5 vs. 11.8%, *p* = 0.049, respectively). An 85% of mothers stated that their breastfeeding experience was different from what they would have expected, blaming for this discrepancy the occurrence of difficulties during breastfeeding and the complexity of breastfeeding itself (50%), pain experience (8%), being dependent from the baby (6%), and breastfeeding failure (11%). A total of 25% of mothers, however, reported they found breastfeeding to be a much more positive experience than what they had expected.

**Conclusion:** Breastfeeding care should include a tailored emotional support of first time-mothers in addition to the implementation of their breastfeeding knowledge and skills.

## Introduction

Overwhelming evidence indicates breastfeeding as infants' normative and unequaled feeding due to the dose-dependent positive impact on their physical and cognitive outcomes (1–3). Accordingly, exclusive breastfeeding is recommended for the first six months and breastfeeding should be continued until at least 2 years of age (4). However, although breastfeeding initiation rates are high, early discontinuation is common in different countries (5, 6).

Research aimed to optimize breastfeeding promotion and support has greatly focused on the biomedical aspects of lactation, whereas the unique emotional breastfeeding experience has been poorly explored (7). Indeed, the breastfeeding process has been recognized as complex and challenging and mothers experience several emotions that shape their breastfeeding experience (8, 9). Mothers are more likely to be satisfied with their breastfeeding experience by 12 months of infants' age when they have experienced positive emotions at 2 months post-delivery (10). In turn, a positive breastfeeding experience favors breastfeeding (11) whereas a previous negative breastfeeding experience may lead to a fear of breastfeeding a next child (12). Furthermore, an early breastfeeding discontinuation may disrupt maternal breastfeeding expectations and/or cause a “breastfeeding grief,” which could undermine maternal emotional well-being and mother-infant relationship (13–15).

First-time mothers have been reported to benefit from a positive breastfeeding experience in terms of growing confidence as a parent (16). This finding is even more important when taking into account that parity is a widely acknowledged determinant of breastfeeding outcome, with first-time mothers being at higher risk for earlier cessation than multiparous ones (17). Moreover, rates of depressive symptoms, anxiety, and sadness during the postpartum period are higher among first-time mothers when compared with multiparas (18). The occurrence of these mental issues could interfere with optimal breastfeeding outcomes (19).

A deeper investigation of maternal breastfeeding experience complexity has thus been advocated in order to develop tailored interventions aimed to promote and support breastfeeding (7). The present study aimed to investigate the emotional breastfeeding experience during the first three months of lactation in a cohort of first-time mothers.

## Materials and Methods

We conducted a prospective, observational study in the neonatal ward of Fondazione IRCCS Ca' Granda Ospedale Maggiore Policlinico in Milan, Italy, from February to June 2019. The hospital provides Level III neonatal care, covering around 6,000 deliveries per year. The study was approved by the institutional Ethical Committee. Written informed consent was signed by the mothers and by both parents for collection of infants' neonatal data.

Enrollment took place at hospital discharge. Inclusion criteria were having delivered singleton, healthy, term (gestational age ≥ 37 weeks) newborns with birthweight ≥2,500 g, being primiparous, exclusively breastfeeding, and Italian-speaking so that no language barriers could lead to inaccurate responses. Multiparous mothers, twin pregnancies, and mothers whose newborns were admitted to the Neonatal Intensive Care Unit and/or had developed any disease that could negatively interfere with breastfeeding were excluded. Breastfeeding promotion and support was implemented in all mother-infant dyads throughout the hospital stay according to the 10 Steps to Successful Breastfeeding (20).

Maternal socio-demographic characteristics and basic infants' data were collected at enrollment. Mothers were asked about their breastfeeding intention and were instructed to complete at 3 months after delivery an online questionnaire (Table 1). The questionnaire was developed by a dedicated group of neonatologists, pediatricians, nurses, nutritionists, two International Board Certified Lactation Consultants and one psychologist, with the aim of covering four areas: breastfeeding experience (items 2–4, 10–11), breastfeeding difficulties (items 5–7), breastfeeding support (items 8, 9), and intention to breastfeed again in a following pregnancy (item 12). The questionnaire included four open-ended questions (Table 1). With regard to the area concerning the maternal breastfeeding experience, the investigated emotions included primary ones, defined as inborn affective states (fear, sadness, anger, joy) and secondary ones, defined as emotions requiring a higher cognitive process to arise (weariness, concern, disappointment, fulfillment, gratification serenity) (21). At 3 months of infants' age, mothers were contacted by phone in order to remind them to access and complete the online questionnaire. A correct understanding of question 10, which was related to the maternal feelings experienced, was promoted during the reminder call, when each term was explained thoroughly according to the description of the meaning inserted in the questionnaire. Mothers were also instructed to indicate all the feelings that applied to their experience when filling in the questionnaire. Mothers were also asked to indicate the mode of feeding during the previous 24 h: exclusive breastfeeding, complementary breastfeeding and exclusive formula feeding (item 1) (4). Exclusive breastfeeding was defined as the administration of no other food or drink, not even water, except breast milk; complementary breastfeeding as the administration of both breast milk and formula milk and/or other liquids or non-dairy foods; exclusive formula feeding as the sole administration of formula milk (22).

**Table 1:** Online questionnaire accessed by mothers at 3 months post-delivery.

***N***	**Items**	**Answers**
1	Infants' mode of feeding during the previous 24 h	- Exclusive breastfeeding (infant consumes only breast milk and no other food or drink, not even water) - Complementary breastfeeding (infant consumes breast milk but alsoformula milk and other liquid or non-dairy foods) - Formula feeding (infant consumes only formula milk)
2	How would you describe your breastfeeding experience?	- Very positive - Moderately positive - Mixed positive and negative - Slightly negative - Negative
3	Please, try to explain the reason	Open question
4	In which of these situations do you now recognize yourself?	- I breastfeed and I like it - I breastfeed although I am encountering difficulties since I believe it is right - I would have liked to continue breastfeeding (exclusively or not exclusively) but it has not been possible - I do not breastfeed anymore because it has been difficult; had it not been difficult, I would have kept on breastfeeding - I have chosen not to breastfeed anymore
5	Did you encounter any difficulties in breastfeeding?	- Yes - No
6	If yes, could you specify which difficulty you encountered?	Open question
7	How old was your infant when you began experiencing breastfeeding difficulties?	- Days - Months
8	Did you receive breastfeeding support from health professionals during hospital stay and after discharge?	- Yes - No
9	Which kind of support would have you liked to receive but you did not?	Open question
10	Which of the following feelings did you experience in these 3 months? (multiple answers possible)	- Joy (complete state of bliss) - Fulfillment (pleasant feeling of happiness for having achieved what one desires) - Serenity (peace and calmness) - Satisfaction (contentement regarding how breastfeeding is going) - Emotional exhaustion (chronic state of emotional and physical depletion) - Concern (uncomfortable but controllable feeling of worry) - Fear (unpleasant and disturbing anticipation of something bad happening to them or their infants) - Sadness (pervasive gloominess) - Anger (strong feeling of annoyance toward current situation) - Disappointment (non - fulfillment of hopes or expectations)
11	During these 3 months of breastfeeding experience which differences did you find with respect to what your expectations were?	Open question
12	Would you breastfeed again?	- Yes - No

## Statistical Analysis

A descriptive analysis was performed with mean values and standard deviations and absolute and relative frequencies. Breastfeeding intention was reported as median, range and interquartile range.

Answers to the four open-ended questions of the questionnaire were classified and analyzed using Thematic Coding as described by Gibbs, 2007 (23). To each theme emerging from the answers, a specific number was assigned for statistical analysis purposes.

For analysis, the feelings experienced by the mothers were classified as positive (joy, fulfillment, serenity, satisfaction) and negative (emotional exhaustion, concern, fear, sadness, anger, disappointment).

The chi-squared test was used to assess the association between the feelings experienced during breastfeeding and feeding outcomes at 3 months. The association between the positive and negative feelings experienced and mothers' basic characteristics was also assessed. All statistical analyses were conducted using SPSS (version 25, SPSS, Inc., Chicago, IL) and statistical significance was set at the α = 0.05 level.

## Results

Of the 1,483 mothers that had delivered during the study period, 437 were eligible for the study and 421 were enrolled whereas 16 refused to take part to the study. Of the enrolled mothers, 273 (65%) accessed the online questionnaire and completed the study whereas 148 could not be reached by phone and did not complete the online questionnaire at 3 months after delivery. No significant difference among the basic characteristics of the mother-infant pairs that completed the study and those of the mother-infant pairs that did not complete it was found (Table 2). The majority of the enrolled mothers were married or cohabitant, with a high level of education, and underwent a spontaneous delivery. At 3 months post-delivery exclusive breastfeeding was reported by a 66% of mothers, a 19% reported complementary feeding, and a 15% of mothers reported formula feeding. The median breastfeeding intention was 6 months (range = 22 months and interquartile range = 6 months). Breastfeeding experience was described as very positive or moderately positive in 62% of cases, whereas 26% of the mothers reported breastfeeding as a mixed positive and negative experience, and 12% rated their experience as negative or slightly negative. The most frequent reasons reported by the mothers that described their breastfeeding experience as positive were a satisfied desire of protecting the baby and bonding with him/her (26%), breastfeeding being comfortable and cheap (11%), breastfeeding being an adequate food for the baby while allowing the mother-infant dyad to feel good (9%), breastfeeding being a unique and wonderful experience (8%), breastfeeding making them feel calm and relaxed (10%). The most frequent reasons reported by the mothers that had described their breastfeeding experience as negative were fatigue and challenges posed by breastfeeding, particularly at the beginning (29%), breast problems (18%), perception of limited milk supply (16%), latching difficulties (7%), being “unsure” about breastfeeding (5%), infant's growth faltering (4%), frequent nursing (4%), feeling not adequately supported (4%), maternal health problems (2%).

**Table 2:** Basic characteristics of the enrolled mother-infant pairs.

	**Mother-infant pairs who completed the study (*n* = 273)**	**Mother-infant pairs that did not complete the study (*n* = 148)**
	**Mean (SD)**	**Mean (SD)**
**Mothers**		
Maternal age (years)	34.5 (4.6)	34.5 (4.9)
Marital status	*N* (%)	*N* (%)
Married	145 (53.1)	76 (51.4)
Cohabitant	123 (45.1)	71 (47.9)
Single	4 (1.4)	0 (0)
Divorced	1 (0.4)	1 (0.7)
Maternal educational level		
≤13 years	65 (23.8)	38 (25.7)
>13 years	208 (76.2)	110 (74.3)
Vaginal delivery	174 (63.7)	93 (62.8)
**Infants**		
Gestational age (weeks)	39.4 ± 1	39.4 ± 1
Birth weight (g)	3,322 ± 358	3,360 ± 323
Length (cm)	50.1 ± 1.7	50.0 ± 1.5
Head circumference (cm)	34.3 ± 1.4	34.2 ± 1.5

With regard to the question: “In which of these situations do you now recognize yourself?” (item 4), a 51% of the mothers declared they were breastfeeding and liking it and a 29% stated they were breastfeeding because it was the right thing to do even though they had encountered difficulties. In 14% of cases, mothers would have liked to breastfeed longer, either exclusively or not, but it was not possible. Breastfeeding was stopped due to the occurrence of difficulties or following a personal choice in 6% of cases (Table 3).

**Table 3:** Frequency of answers to item *n*. 4.

**In which of these situations do you now recognize yourself?**	***N* (%)**
I breastfeed and I like it	140 (51)
I breastfeed although I encounter difficulties because I believe it is right	78 (29)
I would have liked to continue breastfeeding (exclusively or not exclusively) but it has not been possible	38 (14)
I do not breastfeed anymore because it has been difficult; had it not been difficult, I would have kept on breastfeeding	15 (5)
I have chosen not to breastfeed anymore	2 (1)

A total of 218 (80%) mothers experienced difficulties during breastfeeding and reported their occurrence in a time range comprised between 8.9 and 19.9 days post-delivery. No difference was found in the occurrence of breastfeeding difficulties according to mode of delivery. The difficulties most frequently reported were pain and fatigue (35%), cracked nipples (25%), perception of a low milk supply (19%), breast problems (17%), sucking difficulties (16%), factors related to the infant (7%) and infant's failure to thrive (4%). Mothers were supported by healthcare professionals both during hospital stay and after discharge in 89% of cases and were satisfied with their advices in 55% of cases. A total of 17% of the mothers would have liked to receive a more tailored support, 13% would have needed more support during hospital stay and 7% after discharge. Psychological support, education on breastfeeding technique, and a better education during pregnancy were advocated by 5%, 4% and 3% of mothers.

Mothers reported to have experienced positive and negative feelings during the 3 months post-delivery. Positive feelings were experienced by the majority of the mothers (Table 4). With regard to the negative feelings, emotional exhaustion was the most frequently reported followed by concern and fear. Sadness, anger and disappointment were reported by 27% of the mothers (Table 4).

**Table 4:** Positive and negative feelings experienced by the mothers.

**Positive feelings**	***N* (%)**	**Negative feelings**	***N* (%)**
Joy	269 (98.5)	Emotional exhaustion	157 (57.5)
Fulfillment	264 (96.7)	Concern	112 (41.0)
Serenity	261 (95.6)	Fear	55 (20.1)
Gratification	267 (97.8)	Sadness	35 (12.8)
		Anger	20 (7.3)
		Disappointment	19 (7.0)

Concern was experienced by the mothers who delivered by cesarean section in a higher percentage of cases than mothers who delivered vaginally (56.3 vs. 43.7, *p* = 0.04), who, in turn, experienced sadness in a lower percentage of cases (45.7 vs. 54.3, *p* = 0.023). No difference was found among the experience of fear, emotional exhaustion, disappointment, anger or any of the positive feelings and mothers' basic characteristics.

The mothers who experienced anger during breastfeeding showed a significant higher formula feeding rate at 3 months than those who did not. The experience of concern, fear and sadness during breastfeeding tended to be associated with a higher formula feeding rate at 3 months but did not reach statistical significance (Table 5).

**Table 5:** Associations between maternal negative feelings and feeding outcomes at 3 months.

**Feeding Outcomes**	**Yes (%)**	**No (%)**	***P***
	**Emotional exhaustion**		
Exclusive breastfeeding	63.1	69.0	
Complementary breastfeeding	21.0	16.3	
Formula feeding	15.9	14.7	0.555
Breastfeeding (exclusive and complementary)	84.1	85.3	
Formula feeding	15.9	14.7	0.774
	**Concern**		
Exclusive breastfeeding	58.0	70.8	
Complementary breastfeeding	21.5	17.4	
Formula feeding	20.5	11.8	0.064
Breastfeeding (exclusive and complementary)	79.5	88.2	
Formula feeding	20.5	11.8	0.049
	**Fear**		
Exclusive breastfeeding	60.0	67.0	
Complementary breastfeeding	14.5	20.2	
Formula feeding	25.5	12.8	0.061
Breastfeeding (exclusive and complementary)	74.5	87.2	
Formula feeding	25.5	12.8	0.021
	**Sadness**		
Exclusive breastfeeding	51.4	67.7	
Complementary breastfeeding	20.0	18.9	
Formula feeding	28.6	13.4	0.056
Breastfeeding (exclusive and complementary)	71.4	86.6	
Formula feeding	28.6	13.4	0.021
	**Anger**		
Exclusive breastfeeding	45.0	67.2	
Complementary breastfeeding	15.0	19.4	
Formula feeding	40.0	13.4	0.006
Breastfeeding (exclusive and complementary)	60.0	86.6	
Formula feeding	40.0	13.4	0.005
	**Disappointment**		
Exclusive breastfeeding	47.4	66.9	
Complementary breastfeeding	26.3	18.5	
Formula feeding	26.3	14.6	0.204
Breastfeeding (exclusive and complementary)	73.7	85.4	
Formula feeding	26.3	14.6	0.186

When pooling together exclusive and complementary breastfeeding mothers, those who experienced fear, sadness, anger and concern during breastfeeding reported a significantly higher rate of formula feeding at 3 months than those who did not (Table 5).

No association was found between the experience of positive feelings during breastfeeding and exclusive formula feeding at 3 months. An 85% of mothers stated that their breastfeeding experience was different from what they would have expected. The mothers who found it more difficult than expected, blamed for this discrepancy the occurrence of difficulties during breastfeeding and the complexity of breastfeeding itself (50%), pain experience (8%), being dependent from the baby (6%), and breastfeeding failure (11%). A total of 25% of mothers, however, stated they found breastfeeding to be a much more positive experience than what they had expected. Three mothers did not specify if and how reality differed from their expectations. Mothers declared their intention to breastfeed again in a following pregnancy in 95% of the cases.

## Discussion

The results of our study indicate that, although the majority of mothers enjoyed a positive breastfeeding experience, almost 40% reported either mixed or negative emotions, thus reflecting that mothers may face several challenges and difficulties during the complex breastfeeding process. Consistently, although the rate of exclusive breastfeeding at 3 months in the present study is even higher than national data (24), the percentage of mothers formula feeding at 3 months was significantly higher in those who experienced negative feelings, including fear, sadness, anger and concern, than in those who did not. Breastfeeding experience is unique for each mother (7). In the present study, mothers planned a median breastfeeding duration of 6 months and 25% of them reported that their breastfeeding experience turned up to be better than expected. However, it has to be taken into account that most mothers stated their breastfeeding experience was different from their expectations even though almost all of them would breastfeed again. Mothers having delivered by cesarean section experienced concern in a lower percentage of cases than mothers that have delivered vaginally, probably reflecting an adequate breastfeeding support. However, the finding that mothers who delivered by cesarean section experienced sadness in a higher percentage of cases underlines the important role played by mode of delivery in modulating birth experience and maternal feelings ranging from distress to postpartum depression (25).

Taken together, these results indicate a relative lack of emotional support and reassurance to women who experience negative emotions. Accordingly, an additional breastfeeding support, either during hospital stay or after discharge, including a phsycological one was advocated by almost half of the mothers.

Furthermore, mothers appear not to have been adequately forewarned with regard to the difficulties that can arise during breastfeeding and the existence of a gap between maternal expectations and real-life. Of note, regardless of the difficulties, up to nearly 30% of the mothers declared they were breastfeeding because they thought it was right for the baby, whereas only a very limited percentage of mothers stated they had stopped breastfeeding following a personal choice. These results indicate that mothers may feel societal pressure to breastfeed and mainly focus on the benefits for the baby, breastfeeding being considered as a “fundamental maternal commitment” (9). Within this context, it must be kept in mind that breastfeeding has been considered as an indicator of being a “good mother.” Hence, mothers are motivated to persevere facing obstacles and, if an interruption of breastfeeding occurs earlier than desired, their self-confidence is negatively affected (26). Among the positive emotions, however, mothers reported that breastfeeding was a unique and wonderful experience, that allowed them to protect and bond with their baby, making them feel calm and relaxed. Taken all together, these findings point out the dynamic and complex interplay between breastfeeding and the transition to motherhood.

The results of the present study are consistent with previous data reported by other authors. Leurer et al. (7) investigated maternal breastfeeding experience during the first six months by administering a survey to 551 mothers, with a response rate of 35%. The authors highlighted that mothers, although reporting an overall positive breastfeeding experience, had to deal also with mixed and negative emotions. These were related not only to the occurrence of breastfeeding difficulties, but also to the frustration for experiencing them in doing something that is considered to be natural and easy. The study by Leurer et al. (7) further underlined that the negative emotions were associated also with the uncertainty of adequate milk supply, the experience of pain and discomfort and the feeling of being limited in performing other activities due to the need of staying physically with the baby.

Guttman et al. (27) interviewed 54 mothers within an urban low-income context in the United States. These mothers were overall aware of breastfeeding benefits and experienced guilt when stopping breastfeeding. Forster et al. (9) conducted a survey investigating the breastfeeding experience of 889 mothers, admitted to an Austrailan public tertiary hospital, who reported both positive and negative feelings. They conclude that breastfeeding support should include not only mothers' education on breastfeeding skills and health benefits but also the psychological, cultural and emotional aspects that affect breastfeeding success. The importance of gaining understanding of maternal breastfeeding experience in order to provide adequate emotional support has been further highlighted by Brown et al. (28). They administered a questionnaire to 217 women who had begun breastfeeding but had stopped before their baby was 6 months old. The authors reported that the occurrence of physical difficulties and pain while breastfeeding was associated with depressive symptoms which in turn represent a risk factor for earlier introduction of formula and breastfeeding discontinuation (29).

Palmer et al. (12) demonstrated that a previous negative breastfeeding experience may lead to a fear of breastfeeding, with mothers being less likely to breastfeed a future child. On the other hand, consistently with the findings of the present study, Wouk et al. (10) reported that experiencing positive outcomes during breastfeeding is associated with a lower risk of introducing formula or solid foods by 6 months of infant's age.

Our study has several limitations that should be acknowledged. With a response rate of 65%, there is the risk of responder bias with mothers having a greater interest in breastfeeding completing the questionnaire. Thus, the present findings could not apply to all first-time breastfeeding mothers. Anyhow, when considering cohort studies, it has been suggested that dropout rates up to 50% can be acceptable (30). We collected data through an anonymous questionare and not during an in-person interview. Therefore, social desirability bias may have not affected responses. The strength of the present study is that it focused on first-time breastfeeding mothers who are at higher risk for early breastfeeding discontinuation and the development of adverse mental issues while transitioning to motherhood.

## Conclusions

The present findings may contribute to a deeper understanding of the emotional breastfeeding experience of first-time mothers and indicate the need for health care professionals to take into account not only the biomedical aspects of lactation but also the breastfeeding experience when promoting and supporting breastfeeding initiation and duration. Considering the complex interplay between breastfeeding and transition to motherhood, the implementation of maternal knowledge and skills related to breastfeeding should be accompanied by an adequate tailored emotional support of breastfeeding mothers, including the availability of a counseling service for first-time mothers.

## Data Availability Statement

The datasets generated for this study are available on request to the corresponding author.

## Ethics Statement

The studies involving human participants were reviewed and approved by Fondazione IRCCS CA' Granda Ospedale Maggiore Policlinico Milano Italy. Written informed consent was signed by the mothers and by both parents for collection of infants' neonatal data.

## Author Contributions

MG conceived and designed the study and wrote the first draft of the manuscript. ML, GS, EB, PS, and LZ collected the data and were responsible for database management. AC and DM performed the analysis and contributed to the discussion of the results. GB, LC, MB, LP, GC, and EV contributed to the conception and design of the manuscript and wrote sections of the manuscript. PM and FM provided suggestions concerning the content and concept of the article. All authors critically revised the manuscript and approved the final version.

## Conflict of Interest

The authors declare that the research was conducted in the absence of any commercial or financial relationships that could be construed as a potential conflict of interest.
